# Members of the uncultured bacterial candidate division WWE1 are implicated in anaerobic digestion of cellulose

**DOI:** 10.1002/mbo3.144

**Published:** 2014-02-05

**Authors:** Rim Driss Limam, Rakia Chouari, Laurent Mazéas, Ting-Di Wu, Tianlun Li, Julien Grossin-Debattista, Jean-Luc Guerquin-Kern, Mouldi Saidi, Ahmed Landoulsi, Abdelghani Sghir, Théodore Bouchez

**Affiliations:** 1 Irstea, UR HBAN1 rue Pierre-Gilles de Gennes CS 10030, F-92761, Antony Cedex, France; 2 CEA/Genoscope CNS 2rue Gaston Crémieux, Evry, 91000, France; 3 University of Evry–Val d'EssonneEvry, 91057, France; 4 UR04CNSTN01 “Medical and Agricultural Applications of Nuclear Techniques”, CNSTNSidi Thabet, Ariana, 2020, Tunisia; 5 Faculté des Sciences de Bizerte, Laboratoire de Biochimie et Biologie Moléculaire03/UR/0902, Bizerte, Tunisia; 6 INSERM U.759Orsay, 91405, France; 7 Laboratoire de Microscopie Ionique, Institut CurieOrsay, 91405, France

**Keywords:** Cellulose anaerobic digestion, FISH, SIMSISH, SIP, WWE1 candidate division

## Abstract

Clones of the WWE1 (Waste Water of Evry 1) candidate division were retrieved during the exploration of the bacterial diversity of an anaerobic mesophilic (35 ± 0.5°C) digester. In order to investigate the metabolic function of WWE1 members, a 16S rRNA gene-based stable isotope probing (SIP) method was used. Eighty-seven percent of 16S r rRNA gene sequences affiliated to WWE1 candidate division were retrieved in a clone library obtained after polymerase chain reaction (PCR) amplification of enriched DNA fraction from anaerobic municipal solid waste samples incubated with ^13^C-cellulose, at the end of the incubation (day 63) using a Pla46F-1390R primer pair. The design of a specific WWE1 probe associated with the fluorescence in situ hybridization (FISH) technique corroborated the abundant representation of WWE1 members in our ^13^C-cellulose incubations. Secondary ion mass spectrometry–in situ hybridization (SIMSISH) using an iodine-labeled oligonucleotide probe combined with high-resolution nanometer-scale SIMS (NanoSIMS) observation confirmed the isotopic enrichment of members of WWE1 candidate division. The ^13^C apparent isotopic composition of hybridized WWE1 cells reached the value of about 40% early during the cellulose degradation process, suggesting that these bacteria play a role either in an extracellular cellulose hydrolysis process and/or in the uptake fermentation products.

## Introduction

The environmental benefits of anaerobic digestion are the production of energy-rich biogas and the reduction in the waste emission potential from municipal solid waste. Methane production by microorganisms from anaerobic digestion can be considered as a way to gain safe and sustainable energy (McKendry 2002; Liu et al. 2009). Under methanogenic conditions, the production of methane requires a consortium of a variety of strictly anaerobic microorganisms (Ferry 1997). The microbial communities involved in this process and the impact of operational conditions on their activities have received increasing attention during recent years. In the first step of the anaerobic degradation process, the hetero-fermentative microorganisms reduce cellulose and other complex molecules into volatile fatty acids (VFA), CO_2_ and hydrogen (H_2_). Second, syntrophic bacteria which are acetogens further transform fatty acids (R-COOH) and alcohols (C_n_H_2n+1_OH) into acetate (CH_3_COO^−^), H_2_, and CO_2_. Acetate can also be produced by homoacetogenesis in which hydrogen is used to reduce carbon dioxide to acetate. Finally, CH_3_COO^−^, CO_2_, and H_2_ serve as substrates for methanogenic archaea to produce CH_4_ (Guedon et al. 2002).

Among the organic fractions of municipal waste, cellulosic compounds represent a major part. They are generated from domestic, industrial, and agricultural activities. Indeed, they are much more resistant to digestion than protein, lipid, and sugar fractions. In general, the anaerobic digestion of cellulosic waste using biological conversion is carried out by complex microbial communities responsible for hydrolysis, acidogenesis, acetogenesis, and methanogenesis (Antoni et al. 2007). Because of the highly rigid structure of the cellulose, hydrolysis is a slow and rate-limiting step for the biological conversion of cellulosic waste (Pavlostathis et al. 1988; Schwarz 2001). Under mesophilic conditions, the consortium responsible for cellulose degradation has been reported to include *Clostridium thermocellum*,*Clostridium stercorarium*,*Bacteroides cellulosilvens*, and *Acetivibrio spp*. (Burrell et al. 2004; O'Sullivan et al. 2005; Li et al. 2009).

Cellulose degrading microbes have been studied in various natural and engineered ecosystems by several culture-based methods (Barlaz et al. 1989). However, the microbial phyla capable of degrading lignocellulosic materials that have been isolated so far only represent a minor fraction of the phylogenetic groups widely distributed in the environment (Lynd et al. 2002). Anaerobic cellulolytic microorganisms can be found mainly in the genera *Acetivibrio, Anaerocellum, Butyrivibrio, Caldicellulosiruptor, Clostridium, Eubacterium, Fervidobacterium, Halocella, Spirochaeta, Thermotoga, Fibrobacter*,*and Ruminococcus*. However, the in situ ecophysiology of cellulose degradation in complex microbial ecosystems is poorly documented. For the past 20 years, molecular tools developed and used by microbial ecologists have generated a vast quantity of data, and this information has redefined our vision of prokaryotic diversity. These methods, especially 16S rRNA gene sequence analysis, have also been applied during the exploration of the bacterial diversity of the anaerobic mesophilic digester, and a subdominant bacterial group called WWE1 (Waste Water of Evry 1) was discovered by molecular inventories of the anaerobic digester of Evry (Chouari et al. 2005). It was found that WWE1 bacteria could represent up to 10% of the bacterial microflora (Chouari et al. 2005) and was often observed in anaerobic ecosystems (Chouari et al. 2005; Elshahed et al. 2007; Riviere et al. 2009). A study by massive sequencing allowed Pelletier and colleagues (Pelletier et al. 2008) to obtain a good draft genome of the *Candidatus Cloacamonas spp* belonging to the WWE1 candidate division. Pelletier and colleagues have suggested that WWE1 is a syntrophic amino acid metabolizer because of the presence of genes coding for several hydrogenases, together with five different ferredoxin oxidoreductases, which are typically involved in amino acid fermentation (Pelletier et al. 2008). However, as is the case for many uncultured groups of bacteria, the in situ ecophysiology of the WWE1 group in complex microbial ecosystems is poorly documented. In recent years, a number of techniques allowing microbial ecologists to directly identify a specific metabolic activity of functional groups of microorganisms within communities of uncultured microbes have been proposed. SIP (stable isotopic probing) is a powerful tool for detecting and identifying active members of natural microbial populations involved in the assimilation of an isotopically labeled compound into nucleic acids (Radajewski et al. 2003; Tobino et al. 2011). Moreover, SIP analysis has the distinct advantage of allowing one to specifically harvest genomic DNA from those bacteria consuming a defined substrate (Li et al. 2009). Furthermore, NanoSIMS combined with fluorescence in situ hybridization (FISH) is an approach that can link the phylogeny to function by in situ association of a particular phylotype to substrate uptake (Li et al. 2008). Recent research has indicated that the combination of SIP-NanoSIMS or FISH-NanoSIMS can be used to decipher networks of biogeochemical processes catalyzed by specific groups of microorganisms within complex microbial communities (Behrens et al. 2008; Pett-Ridge and Weber 2012).

The aim of this study was to document the potential functions carried out by the WWE1 group. Batch incubations with municipal solid waste, mature compost of green waste, and cellulose as substrates have been carried out in order to gain more insight into the metabolic function of this group of bacteria. The SIP-16S rRNA gene approach followed by SIMSISH were used here to measure the isotopic composition of WWE1-assimilating labeled carbon originating from ^13^C-cellulose, in municipal solid waste digester samples incubated anaerobically under mesophilic conditions.

## Experimental Procedures

### Batch system incubations

Batch system incubations were carried out in 1000 mL glass bottles closed with a screw cap and septum (Fischer Scientific Bioblock, Illkirch, France). For municipal solid waste batch experiments, 39.6 g of municipal solid waste was reconstituted (in triplicate) as described previously (Qu et al. 2009) and according to the average household waste composition in France (Ademe 1999). For mature compost of green waste batch experiments (in triplicate), the same quantity (39.6 g) of mature compost of green waste was used. For each experimental bottle, 680 mL of leachate originating from a leachate well located in a municipal solid waste landfill (Vert-le-Grand, France) was added and the headspace was purged with helium (He) to obtain less than 0.2% oxygen (O_2_) levels at the beginning of each experiment. These batch systems were incubated anaerobically under mesophilic conditions (35°C). Samples were regularly recovered through the septum with a needle in the different incubations. They were centrifuged (11,000*g* for 10 min at 4°C) and supernatants and pellets were frozen at −20°C until used for chemical analysis and DNA extraction. Chemical analyses were performed separately for each replicate. For molecular studies, only one representative replicate was analyzed.

### ^13^C-labeled cellulose injection

Samples from ^13^C-labeled cellulose incubations already described in Li et al. (2009) were used. Briefly, after the end of the active biogas production period (150 days), the municipal solid waste batch experiments were opened in an anaerobic chamber. Leachate aliquots (50 mL) containing waste residues were dispensed into 120 mL vials. The vials were hermetically sealed with a rubber septum and the headspaces were flushed with helium gas. ^13^C-labeled cellulose (cellulose, 99% in ^13^C, 200 mg, synthesized by *Acetobacter xylinum*; Gagnaire and Taravel 1980) was added to a concentration of 4.75 mg mL^−1^ in different vials (in triplicate) and incubated under a typical mesophilic temperature (35 ± 0.5°C). At the same time, a vial containing unlabeled bacterial cellulose was set up to serve as negative control. Samples were regularly recovered through the septum. They were centrifuged (11,000*g* for 10 min at 4°C) and supernatants and pellets were frozen at −20°C until used for chemical analysis, SIP and SIMSISH.

### Chemical analysis

The biogas composition (CH_4_ and CO_2_) was analyzed periodically by connecting the bottle to a gas chromatograph (*μ*GC CP 4900, Varian) as described in previous research (Qu et al. 2009). The dissolved inorganic carbon (DIC) concentrations were measured using a BIORITECH 700 analyzer (Bioritech, Guyancourt, France). Acetic acid was measured using a Thermo Quest-Trace GC 2000 (Thermo Quest, Milan, Italy) equipped with a flame ionization detector (FID) and a DB-WAXetr Capillary Column as described previously (Qu et al. 2009).

### Measurement of ^13^C-atom% in total inorganic carbon and in acetate

In order to measure the ^13^C-atom% in inorganic carbon, 1 mL of supernatant sample from the ^13^C-cellulose batch was acidified by 200 *μ*L phosphoric acid in a sealed 30 mL flask prefilled with helium. The CO_2_ formed was transferred to a 5 mL vacutainer prior to analysis by gas chromatography–isotopic ratio mass spectrometry (GC-IRMS, Thermo Fisher Scientific, Courtaboeuf, France). The remaining liquid was subjected to acetate ^13^C enrichment measurement by head space gas chromatography–isotopic ratio mass spectrometry (HS-GC-IRMS, Thermo Fisher Scientific). The ^13^C-atom% was calculated according to the formula (^13^C/(^12^C + ^13^C)) × 100%.

### DNA extraction and polymerase chain reaction amplification

For municipal solid waste (day 78) and mature compost of green waste batch experiments (day 58), when the stable methanogenesis phase was started, the total DNA was extracted from pellets using the Powersoil DNA isolation Kit (MoBio Laboratories Inc., Carlsbad, CA). About 0.05–0.2 g of wet pellets was used and processed according to the manufacturer's instructions. For ^13^C-cellulose batch systems, the total DNA extraction and density-gradient ultracentrifugation were performed as described by Li and collaborators (Li et al. 2009). One microgram of total DNA was loaded onto a cesium chloride (CsCl) gradient that was centrifuged at 150,000*g* for 20 h. Gradient fractions of 100 *μ*L were recovered from the bottom of the gradient by pumping water into the top of the tube under constant flow (200 *μ*L min^−1^). DNA quantification (PicoGreen; Invitrogen, Saint Aubin, France) and density measurement (by refractometry) were performed for each fraction in order to define the unlabeled (“light”) and ^13^C-labeled DNA (“enriched fraction”). DNA was isolated from the CsCl by MicroCon YM-10 column (Millipore). Bacterial 16S rRNA genes were amplified by polymerase chain reaction (PCR) using the combination of universal primer 1390R (Univ1390R) (5′-GACGGGCGGTGTGTACAA-3′) (Amann et al. 1995) and Pla46F primer (5′-GGATTAGGCATGCAAGTC-3′) (Neef et al. 1998) resulting in an amplification product of about 1350 bp The PCR amplification profile was performed as follows: initial denaturation at 94°C for 1 min and 30 cycles consisting of denaturation at 94°C for 1 min, primer annealing at 59°C for 1 min, and extension at 72°C for 1.5 min. The final elongation step was extended to 15 min.

### Cloning and sequencing

PCR product cloning was carried out using TOPO TA cloning kit (Invitrogen) into chemically competent *Escherichia coli* top 10 cells, and positive clones were tested according to the manufacturer's instructions. Plasmid extraction and 16S rRNA gene sequence were performed as described in a previous work (Artiguenave et al. 2000). Bacterial 16S rRNA gene sequences were deposited in GenBank under accession numbers JN093309 to JN093512.

### Sequence analysis

The 16S rRNA gene clone sequences were reconstructed with Phrap (http://www.phrap.org/) and assigned to operational taxonomic units (OTUs) using DOTUR (Schloss and Handelsman 2005). A 97% similarity threshold was used for OTU assignment (Goebel and Stackebrandt 1994). For phylogenetic analysis, sequences were imported into the ARB database (Ludwig et al. 2004). All sequences were imported into the ARB database and automatically aligned against the closest related 16S rRNA gene sequences defined by online BLAST results (http://www.ncbi.nlm.nih.gov/BLAST). The resulting alignments were manually checked and when necessary corrected. 16S rRNA gene sequence similarities were determined by using the distance matrix tool of the ARB program package. The Phylogenetic tree was generated by neighbor-joining analyses (Saitou and Nei 1987), with Jukes and Cantor corrections, and a 50% invariance criterion for inclusion of individual nucleotide sequence positions in the treeing analyses. Maximum likelihood (Olsen et al. 1994) and parsimony (Swofford 2003) methods were also used. The statistical significance levels of interior nodes were determined by performing bootstrap analyses based on 100 resampling by the neighbor-joining method.

### Probe design and fluorescent in situ hybridization

An oligonucleotide probe targeting the 16S rRNA gene sequence was used to identify WWE1 cells. The design of a probe targeting new clone sequences was accomplished using the PROBE_DESIGN tool from ARB (Ludwig et al. 2004). A FISH probe was chosen by estimating its accessibility to target sites as described previously (Behrens et al. 2003) and was subsequently validated for specificity using the ProbeCheck database (http://131.130.66.200/cgi-bin/probecheck/probecheck.pl). The new probe was synthesized and labeled at the 5′end with fluorescein isothiocyanate (MWG, Ebersberg, Germany). Theoretical suitable hybridization conditions were estimated (Nakatsu and Forney 1996). Hybridization conditions for a probe targeting WWE1 were further optimized with paraformaldehyde-fixed samples from ^13^C-cellulose batch systems. The formamide concentration for optimum probe stringency was empirically determined by performing a series of FISH experiments. Formamide concentration increments started at 0% and went up to 70% (Schmid et al. 2005). The highest formamide concentration allowing good signal intensity was obtained with a 30% formamide concentration in the hybridization buffer. A combination of the WWE1-specific probe and general Eub338mix probes (a mixture of probes Eub 338I, Eub 338II and Eub 338III) (Daims et al. 1999) was used to confirm that the signals were not artefactual. An inverted Zeiss confocal laser scanning microscope (LSM510-META; Carl Zeiss MicroImaging, Jena, Germany) equipped with three lasers (argon, 488 nm; helium–neon, 543 nm; helium–neon, 633 nm) was used for probe-positive visualization and image acquisition.

### FISH and SIMSISH samples preparation

Samples taken from the ^13^C-cellulose mesophilic incubation and from municipal solid waste batch were pelleted at 11,000*g* for 10 min at 4°C. The pellets were washed once with 1 × phosphate-buffered saline (PBS, Sigma, Lyon, France) and resuspended in 200 *μ*L of 1 × PBS and 600 *μ*L paraformaldehyde 4% (Sigma) as fixative. After 3 h of incubation at 4°C, the tubes were centrifuged (11,000*g*, 10 min) and the pellets were washed once again with 1 × PBS and re-suspended in 500 *μ*L of 1 × PBS and 500 *μ*L of pure ethanol. Fixed cells were stored at −20°C. The hybridization of the iodized probe was performed as described in a previous work (Li et al. 2008).

### NanoSIMS procedure

For NanoSIMS analysis, 1 *μ*L of sample was spread on 7 × 7 mm high-purity silicon chips (Silicon Quest International, San Jose, CA) cleaned with ultrapure water and absolute ethanol. After being dried in a vacuum oven at 55°C overnight, the sample was then introduced into a NanoSIMS-50 instrument (CAMECA, Gennevilliers, France) equipped with a cesium ion source with a local vacuum level less than 7 × 10^−8^ Pa surrounding the sample during analysis. The NanoSIMS observations were analyzed by the procedure described by Li et al. (2008). To ensure accurate determination of the ^13^C content, a thin section of a homogeneous resin sample of which ^13^C isotopic composition had been determined by EA-IRMS, was used as a reference during the measurement sessions. For in situ determination of the isotopic composition, image processing was carried out using ImageJ, Java-based free software (W.S. Rasband, ImageJ, US National Institute of Health, Bethesda, MD, http://rsb.info.nih.gov/ij/, 1997–2006). To define the region of bacterial cells distinguished from the non-cell particles the iodized-oligonucleotidic probe (^127^I) was used in order to identify targeted cells. The colocalization of ^127^I-signal with ^13^C-signal indicated hybridization of the probe and assimilation of the label substrate by the microorganism.

## Results

### WWE1 candidate division members are present in waste anaerobic digestion experiments

**Figure 1:**
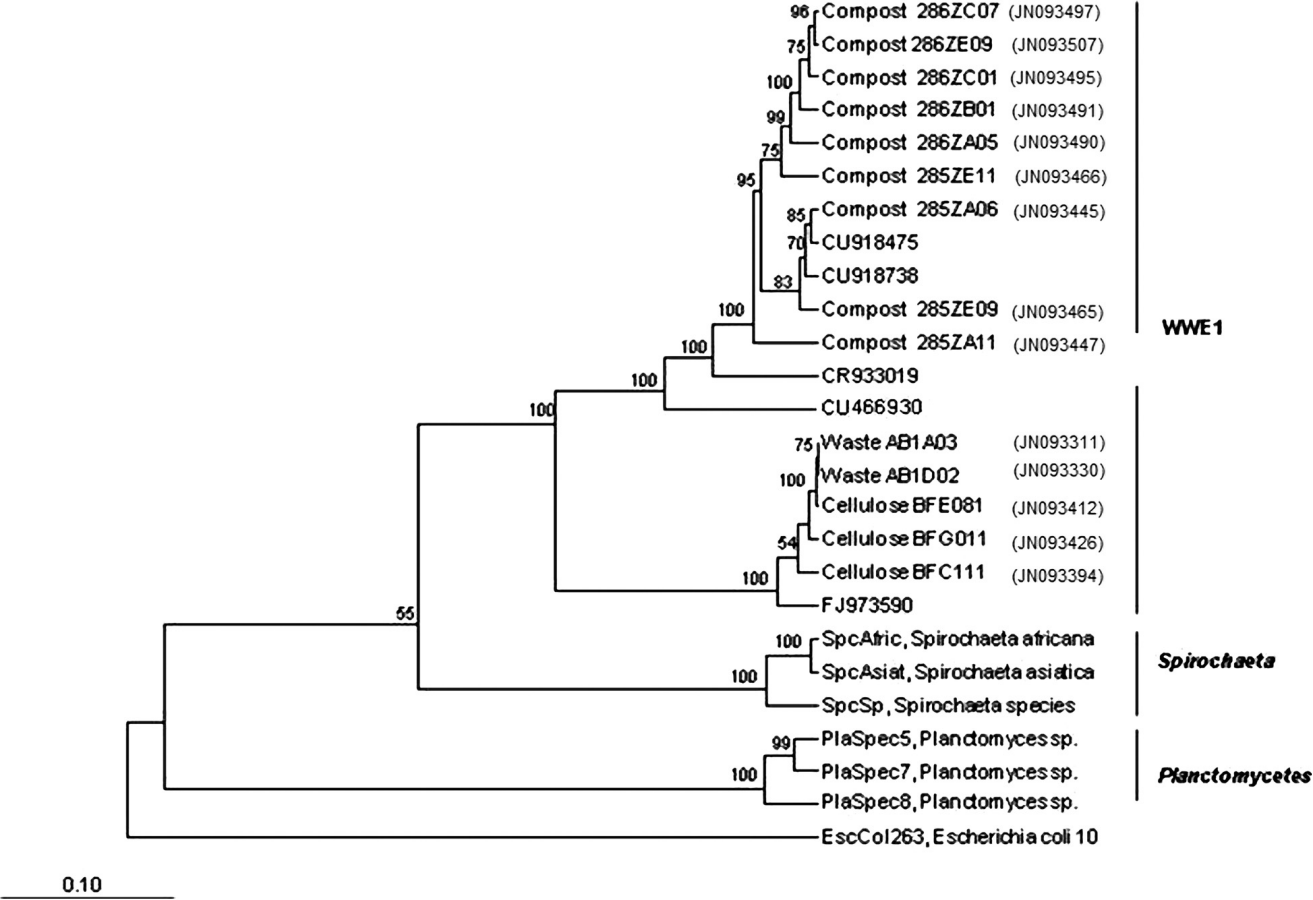
Neighbor-joining phylogenetic tree based on the 16S rRNA gene sequences of representative members of the 14 WWE1 OTUs encountered in mesophilic incubations of mature compost of green waste (labeled “Compost”), waste (labeled “Waste”), and cellulose (labeled “Cellulose”). Sequences from close relatives were included in the analysis (*Spirochaeta Africana, Spirochaeta asiatica*, sequences from *Planctomyces* sp., CU918475, CU918738 [Riviere et al. 2009;], CR933019 [Chouari et al. 2005;] and CU466930 [Pelletier et al. 2008;] FJ973590, [Swan et al. 2010]). Bootstrap values superior to 50% are shown. The tree root was determined using the 16S rRNA sequences of *Escherichia coli* 10 (U00096) as the out-group reference. The scale bar represents a 10% sequence divergence.

Clones of the WWE1 candidate division were initially retrieved during the exploration of the bacterial diversity of an anaerobic mesophilic digester (35 ± 0.5°C) treating sludge (Chouari et al. 2005). However, in such kinds of reactors, the WWE1 candidate division members were subdominants (Chouari et al. 2005). To test if this group could be retrieved during anaerobic digestion of other kind of substrates, genomic DNA was extracted from municipal solid waste and mature compost of green waste batch incubations. It was amplified by PCR with primers Pla46F and 1390R. This primer set was initially described as specific to the *Planctomycetes* phylum. However, it was later recognized as being applicable to a broader phylogenetic range, as it targeted some members of the phylum *Lentisphaera* and WWE1 group (Chouari et al. 2005). A clone library was built from the PCR products of each sample. 16S rRNA sequences were obtained and analyzed. All obtained sequences were affiliated to the domain *Bacteria*. In total, 332 high-quality sequences were obtained. They were further grouped into 14 OTUs. The phylogenetic position of these 14 OTUs is presented on Figure 1.

In the clone library derived from the mature compost of green waste batch experiment, the most abundant sequences were related to the WWE1 candidate division with 47% of the sequences (77 out of 164 sequences). In this sample, the WWE1 group was represented by nine OTUs. These OTUs were closely related to uncultured WWE1 bacterium sequences that were previously detected in an anaerobic sludge digester: CU918475, CU918738 (Riviere et al. 2009), CR933019 (Chouari et al. 2005) and CU466930 (Pelletier et al. 2008) with a sequence identity ranging between 94% and 100% (Fig. 1).

For the waste incubation experiments, the most abundant group of retrieved sequences was related to the WWE1 group with 74% (65 out of 88 sequences). These sequences were represented by two OTUs and showed a sequence identity ranging between 95% and 96% with uncultured bacteria, FJ973590, detected in a hypersaline Salton sea, in California, USA (Swan et al. 2010) (Fig. 1).

### WWE1 candidate division members are retrieved from the heavy DNA fraction of ^13^C-cellulose SIP experiments

Because WWE1 candidate division-related sequences were abundantly retrieved in waste and mature compost of green waste incubation experiments, both containing a high amount of lignocellulosic substrates, the hypothesis of an involvement of WWE1 members in the anaerobic digestion of cellulose was tested using samples from previous SIP experiments that were performed using ^13^C-labeled cellulose (Li et al. 2009). Buoyant-density gradient ultracentrifugation resulted in several distinct DNA-containing density fractions, with a first DNA peak presenting an average density of 1.695 g mL^−1^ on day 7 and a second peak at a density of 1.730 g mL^−1^ on day 21 (Li et al. 2009). On day 63, DNA densities showed a more regularly distributed profile (Li et al. 2009); suggesting a progressive assimilation of labeled carbon by microorganisms.

In the clone library obtained from the enriched DNA fraction of ^13^C-cellulose batch incubation (density of 1.730 g mL^−1^) on day 63, 87% of the sequences were affiliated to the WWE1 group (70 out of 80 sequences) and were assigned to three OTUs. These OTUs showed a sequence similarity (between 95% and 96%) with the uncultured bacteria, FJ973590 (Fig. 1).

### In situ detection of WWE1 candidate division members by FISH

**Figure 2:**
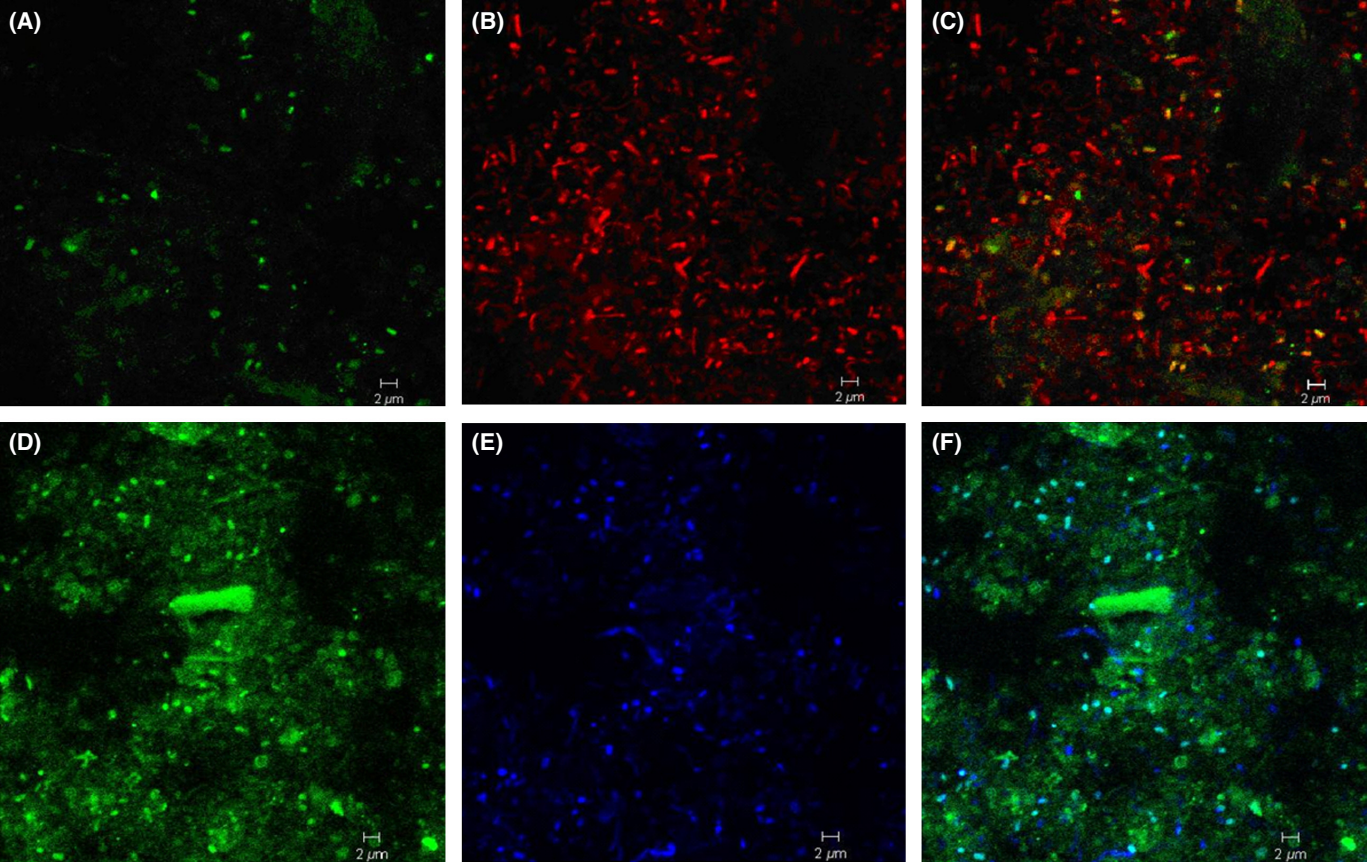
In situ detection of the WWE1 group. Samples from mesophilic waste incubation hybridized with probe S-*-WWE1-408-a-A-19 in green (A) and Eub338mix in red (B), and superimposition of the two images (C). Samples from mesophilic cellulose incubation at day 14 hybridized with oligonucleotide probe S-*-WWE1-408-a-A-19 in green (D), and with probe Eub338mix in blue (E) and superimposition of the two images (F). The scale bar corresponds to 2 *μ*m.

We developed a 16S rRNA oligonucleotide probe specifically targeting the WWE1 group (S-*WWE1-408-a-A-5′-GCTCCGAAAAGCTTCATCG-3′) based on the probe design tool of the ARB program package. A mixture of the new WWE1 specific probe (labeled with fluorescein isothiocyanate), and the bacterial probe EUB338 mix (labeled with monofunctional, hydrophilic sulfoindocyanine dyes Cy3 and Cy5) was applied for screening of the WWE1-related bacteria within the bacterial community of waste and ^13^C-cellulose batch incubations. Because of the presence of autofluorescent particles in some samples, negative controls were always observed in parallel. Moreover, ambiguous fluorescent structures were bleached using the appropriate laser of the CLSM in order to confirm that the observed signals were due to the fluorescent probe. We confirmed the presence of cells from the WWE1 group within the samples of waste and cellulose batch incubations (Fig. 2). FISH observations showed that cells targeted by the WWE1-specific probe had coccobacillus morphology of ∼1 *μ*m diameter and had no apparent particular spatial arrangement pattern. In all samples, members of the WWE1 showed a positive hybridization signal with the EUB338 mix (Fig. 2C and F). Moreover, probe-positive cells that were detected in the samples of ^13^C-cellulose batch incubations were more abundant than those detected in samples of waste batch incubations (Fig. 2) suggesting a relationship between these microorganisms and the anaerobic degradation of cellulose.

### SIMSISH measurement of isotopic composition of WWE1 candidate division members in relation to ^13^C-cellulose degradation

**Figure 3:**
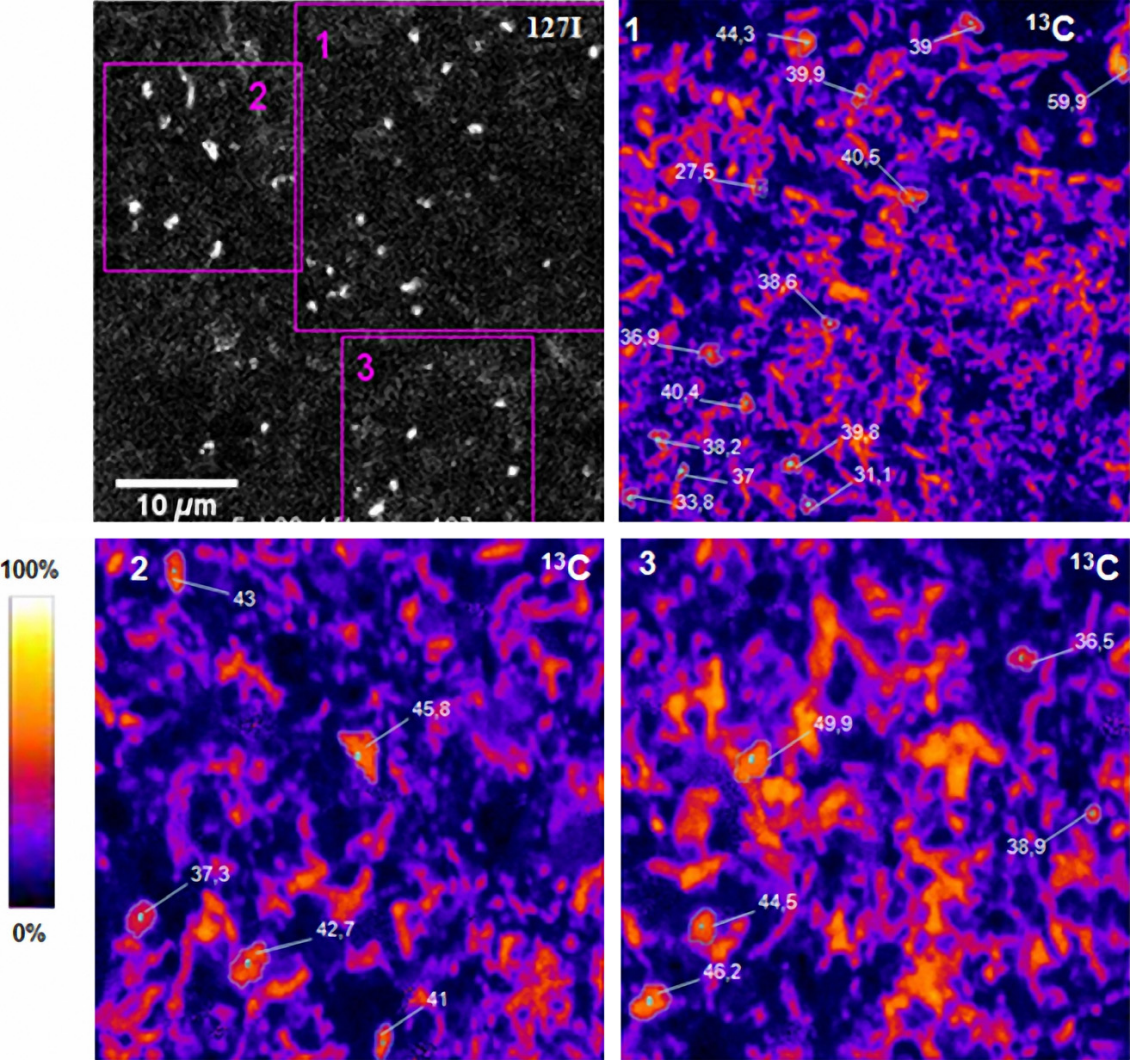
NanoSIMS analysis of WWE1 cells from mesophilic cellulose incubations. The image in black and white represents ^127^I^–^ secondary ion image. Zones 1, 2, 3 are further magnified on images 1, 2, and 3 on which ^13^C-enrichment levels (in atom%) are indicated. The color scale on the left allows to visualize cell isotopic abundance.

In order to verify, at the single-cell level, that the WWE1 group assimilated labeled carbon (^13^C), cell pellet samples of the ^13^C-cellulose mesophilic incubation taken on day 14 (the day on which the most important hybridization signal was obtained) were fixed and analyzed by NanoSIMS (Fig. 3). ^13^C isotopic composition of the cells varied between 31.1 and 59.9%. Many microbes, exhibiting various morphologies, hybridized with probe EUB338-I, were ^13^C-enriched (data not shown). These cells can be divided into two groups. The first group exhibited 45 ± 4.9% of ^13^C isotopic composition. The second group showed 30 ± 5.9% of ^13^C isotopic composition. A major part of the enriched microbes did not exhibit positive hybridization signals with WWE1-specific probe indicating that different community members were actively taking up ^13^C originating from cellulose. Cells hybridized with the iodized WWE1-specific probe (^127^I signal) were, however, also labeled and exhibited an elevated ^13^C composition. The mean value of enrichment WWE1 cells was relatively high (40 ± 5.4%) compared to other microbial community members. This measure indicates that they were among the most active microbes taking up ^13^C atoms originating from cellulose.

**Figure 4:**
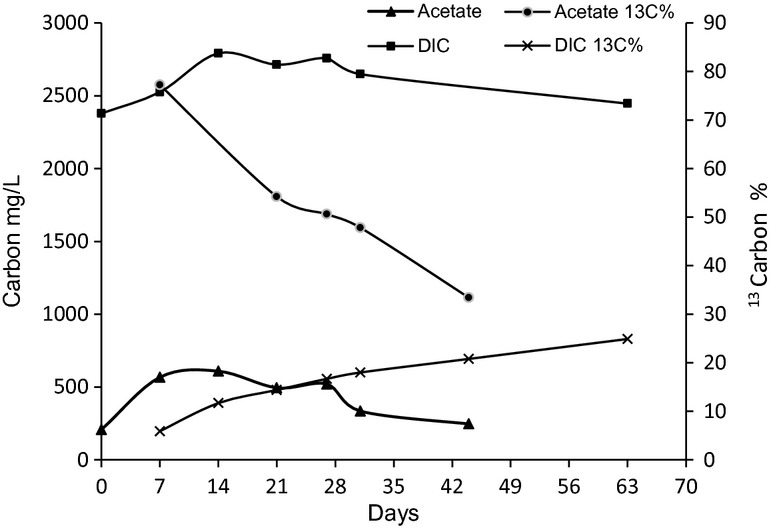
Temporal evolution of acetate and dissolved inorganic carbon (DIC) concentrations and progression of their isotopic composition (^13^C atom%) in mesophilic cellulose incubation experiments.

In order to interpret SIMSISH measurements in relation to the cellulose degradation processes, the fermentation products were analyzed as well as their isotopic composition (Fig. 3). Acetate was the only extracellular fermentation product that could be detected by gas-chromatography analysis. Its concentration increased slightly for 7 days, and then oscillated between 250 and 500 mg L^−1^. In addition to cellulose, acetate and DIC could both represent other ^13^C-carbon source for growth of WWE1 group members. The temporal evolution of the isotopic composition of these compounds was thus followed by GC-C-IRMS (see Material and Methods) (Fig. 4). The acetate ^13^C percentage reached 54% by day 14 which indicated that fully labeled cellulose was then the main source of acetate production. It then continuously decreased to reach 35% on day 44. The DIC ^13^C% followed an opposite trend: its isotopic composition was around 14% by day 14, and then continuously increased to reach 20% on day 44. The high isotopic composition of WWE1 group members at day 14 is therefore not compatible with an autotrophic growth. Their isotopic composition could therefore results only from the uptake of cellulose (which was fully labeled) or an organic substrate derived from hydrolysis and/or fermentation such as acetate.

## Discussion

There are many major groups of *Bacteria* and *Archaea* which are only known from their molecular sequences. The great bulk of microbes have not been cultured yet; and little is known about their function. A main challenge for microbial ecology is to link organisms or groups of organisms to specific functions within their natural environments. In this framework, WWE1 candidate division represents up to 10% of the bacterial microflora and thus could be a subdominant group (Chouari et al. 2005). Therefore, the knowledge of the function and metabolic potentials of its members is of great biotechnological interest.

The Metagenomic functional analyses, whose purpose is to identify activities of interest potentially expressed by uncultured microorganisms, do not allow to infer the reduced set of genes actually expressed which determines the in situ function of the microbial population (Terron-Gonzalez et al. 2013). Consequently, we used in situ methods to gain more knowledge into the WWE1 ecophysiology. We carried out ^13^C-labeled cellulose SIP batch experiments combined with 16S rRNA gene sequence analysis and found that sequences of the WWE1 group were mostly retrieved in the enriched DNA fraction of mesophilic ^13^C-cellulose incubation. These observations suggested that the WWE1 candidate divisions are active members of the microbial community involved in anaerobic cellulose degradation. While SIP constitutes a powerful tool for prescreening active microbial members in situ, the method has some limitations especially the DNA-density shift linked to G+C content which is a potential concern in virtually any DNA SIP experiment (Radajewski et al. 2003; Buckley et al. 2007).

In order to more precisely understand the function of WWE1 group members, we performed in situ observations of WWE1 ^13^C isotopic enrichment at the single-cell level with SIMSISH methodology (Li et al. 2008). We designed and evaluated a specific iodized probe targeting this candidate division. FISH images obtained by means of this probe showed positive signals from the cellulose incubations from day 7 until day 40. Moreover, it should be noted that in the samples from ^13^C-cellulose batch mesophilic incubation, the most abundant hybridization signals were obtained from samples taken at the beginning of the cellulose incubations (day 14), which suggests that the WWE1 group is active during the early phase of cellulose degradation, that is hydrolysis or primary fermentation of hydrolysis products.

Using NanoSIMS to determine in situ cell isotopic composition, we demonstrated ^13^C incorporation in WWE1 cells and revealed that they exhibited an elevated ^13^C composition of about 40%. Therefore, SIP and NanoSIMS results were congruent in indicating that the WWE1 group was actively assimilating ^13^C from cellulose. In addition, we detected some cells which are unrelated to the WWE1 candidate division, exhibiting about 45% and 30% ^13^C composition. This observation showed that these microbes were also implicated in cellulose metabolism. Moreover, a difference in ^13^C composition was observed between cells of the same sample, reflecting a difference in their metabolic status. Cellulose metabolism is a complex process and the high ^13^C composition of different bacterial groups indicates the involvement of various bacterial community members in the process, which is not surprising (Li et al. 2009). The level of ^13^C composition of the WWE1 group in this study suggested that this group was implicated in the assimilation of cellulose or cellulose degradation intermediates derived from cellulose metabolism with an elevated ^13^C composition. Interestingly, hybridized cells targeted by the probe WWE1-408 looked like coccobacillus-shaped cells and were present only in the planktonic phase (Fig. 2D). It should be pointed out that microorganisms specialized in the hydrolysis of cellulose are often localized around cellulose fibers (Burrell et al. 2004; Li et al. 2009). WWE1 group members could therefore play a role in the hydrolysis of cellulose through the release of extracellular cellulase and/or in the fermentation of a highly enriched hydrolysis product resulting from the hydrolytic activity of other bacterial community members.

The measurement of individual cells ^13^C isotopic composition gave a quantitative estimation of the WWE1 contribution to cellulose metabolism. The gas-chromatographic analysis of the samples withdrawn from the cellulose microcosms revealed the presence of acetate as the only volatile extracellular fermentation product. Most of the acetate which was produced during the first 7 days of the incubation came directly from the labeled cellulose as acetate ^13^C% was high (around 78%) on day 7. The enrichment was lower than 100% because around 200 mg L^−1^ of the acetate from a natural isotopic composition were present in the leachate at the beginning of the incubation. The decrease in acetate ^13^C% occurring later on during the incubation may be due to the production of acetate by homoacetogenesis. Previous results from several studies have underlined the role of microorganisms producing acetate by the homoacetogenesis using H_2_/CO_2_ from the mineralized fraction of organic matter (Braun et al. 1981; Hattori et al. 2005). As the ^13^C isotopic composition of the mineralized fraction of carbon (DIC) was low in our incubation, acetate produced through homoacetogenesis should have exhibited a low ^13^C enrichment level. Our results are therefore consistent with an important homoacetogenic activity, which has often been underestimated until now during the fermentation processes, as recently pointed out by Ni et al. (2011). As the WWE1 group members showed higher ^13^C enrichment than DIC ^13^C isotopic composition, it indicates that this group is a heterotrophic group that is not involved in an autotrophic homoacetogenic process. On the basis of our results, we hence argue that members of the WWE1 group have a ^13^C enrichment pattern suggesting their involvement in the fermentation of an intermediate product with an elevated ^13^C composition, possibly a saccharide resulting from cellulose hydrolysis. Our results are therefore consistent with those from Pelletier et al. (2008) who suggested that members of WWE1 are involved in the fermentation of sugars.

## Conclusion

In conclusion, this study first revealed the presence of the WWE1 bacteria in ^13^C-cellulose incubation anaerobic batch systems. Second, in situ methods used to investigate the function of WWE1 members have supported the implication of this group in the anaerobic digestion of cellulose either through an extracellular cellulose hydrolysis process and/or in the fermentation of organic substrates originating from cellulose. For a more detailed investigation of the function of WWE1 bacteria in cellulose metabolic pathway, isotopic methods should be combined with metatranscriptomic and metaproteomics approaches to gain a better knowledge of the specific role of WWE1 bacteria.
